# The Underwriter

**Published:** 1880-02

**Authors:** 


A COPY of the Baltimore *Underwriter* has been placed upon our desk. It is devoted to insurance, as its name imports, and such being the case, it is not surprising that exception should have been taken to our editorial on "Life Insurance" in our last. Had our friend, the editor, viewed the article through his *medical spectacles* only, he would have regarded the suggestions in a *better light.* We have only to add that if we were mistaken in *any* of the statements therein made in regard to the recent orders or associations for life insurance purposes, the error or errors are shared by hundreds of the most intelligent of our fellow-citizens, from *all* the professions and most of the occupations in our midst, including some *life insurance agents.*

